# Peer Review of “COVID-19 Return to Sport: NFL Injury Prevalence Analysis”

**DOI:** 10.2196/38731

**Published:** 2022-04-22

**Authors:** Felicianus Pereira

**Affiliations:** 1 Shaheed Benazir Bhutto Dewan University Karachi City Pakistan

**Keywords:** COVID-19, sport, injury, prevalence, cause, data, statistics, pain, training, practice, physiology, adaptation

*This is a peer-review report submitted for the paper “COVID-19 Return to Sport: NFL Injury Prevalence Analysis”*

## Round 1

### General Comments

This paper [1] has focused on a relevant topic in sports. Athletes are at high risk of injury when they have not performed required training, or do not have a solid training base (as highlighted in the periodization plan).

### Specific Comments

1. Was ethical approval taken to conduct this study?
2. Regarding injuries suffered by athletes, were contact injuries accounted for? (As this is a possible confounding variable in this study.)
